# Wiedemann–Franz Law for Massless Dirac Fermions with Implications for Graphene

**DOI:** 10.3390/ma14112704

**Published:** 2021-05-21

**Authors:** Adam Rycerz

**Affiliations:** Institute for Theoretical Physics, Jagiellonian University, Łojasiewicza 11, PL-30348 Kraków, Poland; rycerz@th.if.uj.edu.pl

**Keywords:** graphene, thermal conductivity, electrical conductivity, Wiedemann–Franz law, Corbino disk

## Abstract

In the 2016 experiment by Crossno et al. the electronic contribution to the thermal conductivity of graphene was found to violate the well-known Wiedemann–Franz (WF) law for metals. At liquid nitrogen temperatures, the thermal to electrical conductivity ratio of charge-neutral samples was more than 10 times higher than predicted by the WF law, which was attributed to interactions between particles leading to collective behavior described by hydrodynamics. Here, we show, by adapting the handbook derivation of the WF law to the case of massless Dirac fermions, that significantly enhanced thermal conductivity should appear also in few- or even sub-kelvin temperatures, where the role of interactions can be neglected. The comparison with numerical results obtained within the Landauer–Büttiker formalism for rectangular and disk-shaped (Corbino) devices in ballistic graphene is also provided.

## 1. Introduction

Soon after the advent of graphene, it became clear that this two-dimensional form of carbon shows exceptional thermal conductivity, reaching the room temperature value of ∼5000 W/m/K [1], being over 10 times higher than that of copper or silver [2]. Although the dominant contribution to the thermal conductivity originates from lattice vibrations (phonons), particularly these corresponding to out-of-plane deformations [3,4] allowing graphene to outperform more rigid carbon nanotubes, the electronic contribution to the thermal conductivity ($\kappa_{\mathrm{el}}$) was also found to be surprisingly high [5] in relation to the electrical conductivity (σ) close to the charge-neutrality point [6]. One can show theoretically that the electronic contribution dominates the thermal transport at sub-kelvin temperatures [7], but direct comparison with the experiment is currently missing. Starting from a few kelvins, up to the temperatures of about T≲80 K, it is possible to control the temperatures of electrons and lattice independently [5], since the electron–phonon coupling is weak, and to obtain the value of $\kappa_{\mathrm{el}}$ directly. Some progress towards extending the technique onto sub-kelvin temperatures has been reported recently [8].

The Wiedemann–Franz (WF) law states that the ratio of $\kappa_{\mathrm{el}}$ to σ is proportional to the absolute temperature [9]

(1) $$\frac{\kappa_{\mathrm{el}}}{\sigma }=LT,$$

where the proportionality coefficient L is the Lorentz number. For ideal Fermi gas, we have

(2) $$L=L_{0}\equiv \frac{\pi^{2}}{3}{\left(\frac{k_{B}}{e}\right)}^{2}≃2.443\times 10^{-8}\;W\cdot \Omega \cdot K^{-2}.$$

For metals, Equation (1) with $L\approx L_{0}$ (2) holds true as long as the energy of thermal excitations $k_{B}T\ll \varepsilon_{F}$, with $\varepsilon_{F}$ being the Fermi energy. Moreover, in typical metals close to the room temperature $\kappa_{\mathrm{el}}\gg \kappa_{\mathrm{ph}}$, with $\kappa_{\mathrm{ph}}$ being the phononic contribution to the thermal conductivity, and even when approximating the Lorentz number as $L\approx (\kappa_{\mathrm{el}}+\kappa_{\mathrm{ph}})/\sigma T$ one restores the value of $L_{0}$ (2) with a few-percent accuracy.

In graphene, the situation is far more complex, partly because $\kappa_{\mathrm{el}}\ll \kappa_{\mathrm{ph}}$ (starting from few Kelvins) but mainly because unusual properties of Dirac fermions in this system. Experimental results of Crossno et al. [5] show that the direct determination of $\kappa_{\mathrm{el}}$ leads to $L/L_{0}=10-20$ for T=50−75 K near the charge-neutrality point. Away from the charge-neutrality point, the value of $L\approx L_{0}$ is gradually restored [10]. In addition, the Lorentz number is temperature-dependent, at a fixed carrier density, indicating the violation of the WF law.

High values of the Lorentz number ($L/L_{0}>10$) were observed much earlier for semiconductors [11], where the upper limit is determined by the energy gap (Δ) to temperature ratio, $L_{\max}\approx {\left(\Delta /2eT\right)}^{2}$, but for zero-gap systems strong deviations from the WF law are rather unexpected. Notable exceptions are quasi one-dimensional Luttinger liquids, for which $L/L_{0}>10^{4}$ was observed [12], and heavy-fermion metals showing $L<L_{0}$ [13].

The peak in the Lorentz number appearing at the charge neutrality point for relatively high temperatures (close to the nitrogen boiling point) can be understood within a hydrodynamic transport theory for graphene [14,15,16], which can be regarded as adaptation of a universal theory of interacting, themalizing physical systems to this specific material. Hydrodynamic theory also allows one to design novel terahertz devices in graphene [16], supplementing earlier studies in this direction [17,18]. However, it is worth stressing that, for clean samples and much lower temperatures, where the ballistic transport prevails, one may still expect Lorentz number peaks with the maxima reaching $L_{\max}/L_{0}\approx 2-3$ and the temperature-dependent widths.

In this paper, we show how to adapt the handbook derivation of the WF law [9] in order to describe the violation of this law due to peculiar dispersion relation and a bipolar nature of graphene. The quantitative comparison with the Landauer–Büttiker results is also presented, both for toy models of the transmission-energy dependence, for which closed-form formulas for L are derived, and for the exact transmission probabilities following from the mode-matching analysis for the rectangular [19,20,21] and for the disk-shaped [22,23] samples.

The remainder of the paper is organized as follows. In Section 2, we recall the key points of the WF law derivation for ideal Fermi gas, showing how to adapt them for massless fermions in graphene. In Section 3, the Landauer–Büttiker formalism is introduced, and the analytical results for simplified models for transmission-energy dependence are presented. The Lorentz numbers for mesoscopic graphene systems, the rectangle, and the Corbino disk, are calculated in Section 4. The conclusions are given in Section 5.

## 2. Wiedemann–Franz Law for Ideal Fermi and Dirac Gases

### 2.1. Preliminaries

**Figure 1:**
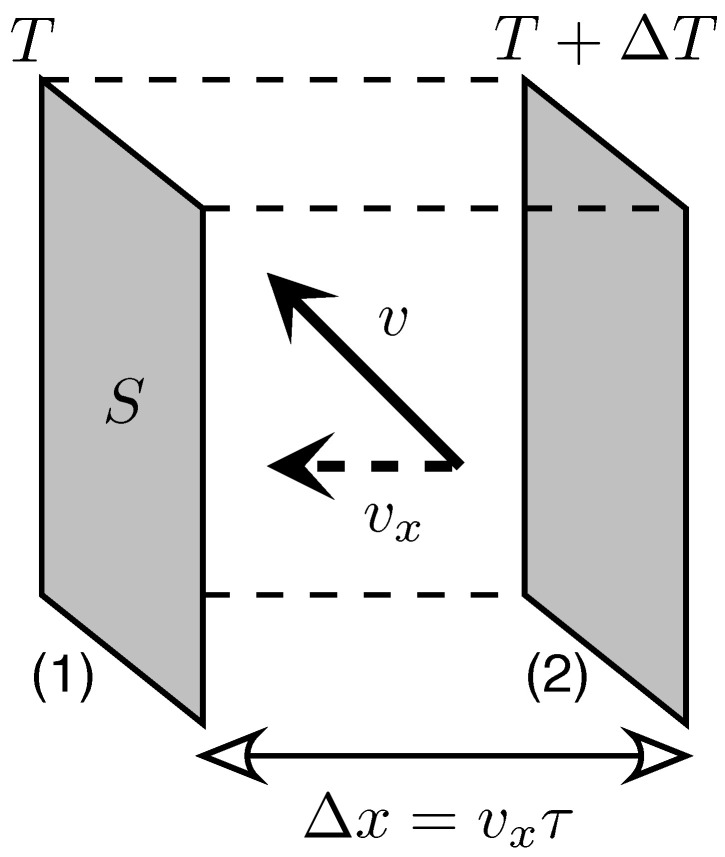
Relation among the thermal conductivity (κ), heat capacity per unit volume (*C*), average particle velocity (*v*), and the mean-free path (*ℓ*). The non-equilibrium heat flow occurs between Interfaces (1) and (2), with local temperatures *T* and T+ΔT, separated by a distance $\Delta x=v_{x}\tau$ (with $v_{x}$ the mean velocity in *x* direction and τ the relaxation time) and can be quantified by ΔQ=CSΔxΔT. The corresponding thermal conductivity is $\kappa =\Delta Q{\left(S\tau \Delta T/\Delta x\right)}^{-1}=Cv_{x}^{2}\tau$. Substituting $v_{x}^{2}=v^{2}/d$ and ℓ=vτ, we obtain Equation (3) in the main text.

The derivation of the WF law for metals [9] starts from the relation between thermal conductivity of a gas with its heat capacity per unit volume (*C*) derived within kinetic theory of gases [24], which can be written as

(3) $$\kappa =\frac{1}{d}\;Cv\;\ell ,$$

where d=1,2,3 is the system dimensionality, *v* is a typical particle velocity, and *ℓ* is the mean-free path (travelled between collisions with boundaries or other particles). Figure 1 presents the key points necessary to obtain Equation (3). It is worth noticing that the definition of *C* in Equation (3), used instead of a familiar specific heat (per unit mass), allows generalizing the reasoning onto the massless particles easily.

Next, the electrical conductivity in Equation (1) is expressed via the Drude formula

(4) $$\sigma =\frac{ne^{2}\ell }{m_{★}v},$$

where n=N/V is the carrier density (to be redefined later for a bipolar system containing electrons and holes) and $m_{★}$ is the carrier effective mass. We skip here the detailed derivation of Equation (4), which can be found in [9]; we only mention that it follows from Ohm’s law in the form j=σE, with j the current density and E the electric field, supposing that carriers of the ±e charge and the $m_{★}$ mass accelerate freely during the time τ=ℓ/v [with the symbols *ℓ* and *v* the same as in Equation (3)]. This time, a generalization for massless particles is more cumbersome; we revisit this issue in Section 2.3.

The system volume, referred to in the definitions of *C* and *n*, can be denoted as $V=L^{d}$, with *L* being linear dimension of a box of gas. In SI units, the dimension of *C* is J/(m${}^{d}\cdot$K), and the unit of thermal conductivity is

(5) $$\left[\;\kappa \;\right]=\frac{W}{m^{d-2}\cdot K}\;.$$

Similarly, the unit of electrical conductivity is

(6) $$\left[\;\sigma \;\right]=\frac{1}{m^{d-2}\cdot \Omega }\;.$$

In turn, the unit of length (m) vanishes in the κ/σ ratio occurring in Equation (1) and the WF law remains valid for arbitrary *d* (provided that the suppositions given explicitly in Section 2.2 are satisfied). Unfortunately, in the literature on graphene, σ is commonly specified in $\Omega^{-1}$ (≡*S*), as follows from Equation (6) for d=2, but the values of κ are reported in W/m/K, as for d=3 [2]. Such an inconsistency can be attributed to the fact that, for the thermal conductivity of multilayer graphene, linear scaling with the number of layers remains a reasonable approximation [25], but the behavior of electrical conductivity is far more complex [26,27] even for bilayers [28].

### 2.2. The Fermi Gas in Metals

The calculation of *C* in Equation (3) employs the free Fermi gas approximation for electrons in a metal. In this approximation, one assumes that leading contributions to thermodynamic properties originate from a thin layer around the Fermi surface. For instance, a contribution to the internal energy can be written as

(7) $$\begin{array}{cc} \Delta U_{\mathrm{el}} & =\int_{\varepsilon_{F}-\Lambda }^{\varepsilon_{F}+\Lambda }d\varepsilon D\left(\varepsilon \right)f\left(\varepsilon \right)\varepsilon \\ \; & \approx \mathrm{const}.+2D\left(\varepsilon_{F}\right){\left(k_{B}T\right)}^{2}\int_{0}^{\infty }dx\frac{x}{e^{x}+1}, \end{array}$$

where $\varepsilon_{F}$ is the Fermi energy, 2Λ is the relevant energy interval considered ($\varepsilon_{F}\gg \Lambda \gg k_{B}T$), D(ε) is the density of states per unit volume (i.e., the number of energy levels lying in the interval of ε,⋯,ε+dε is VD(ε)dε), and f(ε) is the Fermi–Dirac distribution function

(8) $$f\left(\varepsilon \right)=\frac{1}{e^{\left(\varepsilon -\mu \right)/k_{B}T}+1}.$$

In a general case, the chemical potential in Equation (8) is adjusted such that the particle density

(9) $$n\left(\mu \right)=\int_{0}^{\infty }d\varepsilon D\left(\varepsilon \right)f\left(\varepsilon \right)$$

takes a desired value n(μ)≡n, defining the temperature-dependent chemical potential μ=μ(T). Here, the constant-density of states approximation, $D\left(\varepsilon \right)\approx D\left(\varepsilon_{F}\right)$ for $\epsilon_{F}-\Lambda ⩽\epsilon ⩽\epsilon_{F}+\Lambda$ imposed in the rightmost expression in Equation (7), is equivalent to $\mu \approx \varepsilon_{F}$ [29].

The definite integral in Equation (7) is equal to

(10) $$\int_{0}^{\infty }dx\frac{x}{e^{x}+1}=\frac{1}{2}\zeta \left(2\right)=\frac{\pi^{2}}{12},$$

where the Riemann zeta function,

(11) $$\zeta \left(z\right)=\sum_{p=1}^{\infty }\frac{1}{p^{-z}},\;\;\;\;\;\;\mathrm{Rez}>1,$$

is introduced to be used in forthcoming expressions.

Differentiating $\Delta U_{\mathrm{el}}$ (7) over temperature, one gets approximating expression for the electronic heat capacity

(12) $$C_{\mathrm{el}}\approx \frac{\pi^{2}}{3}D\left(\varepsilon_{F}\right)k_{B}^{2}T.$$

In fact, the factor of $\pi^{2}/3$ in Equation (12) is the same as the one appearing in the Lorentz number $L_{0}$ (2), which is shown in a few remaining steps below.

For an isotropic system with parabolic dispersion relation

(13) $$\varepsilon_{k}=\frac{\hbar^{2}k^{2}}{2m_{★}},$$

bounded in a box of the volume $V=L^{d}$ with periodic boundary conditions, the wavevector components $k=(k_{i})$ take discrete values of $k_{i}=0,\pm \frac{2\pi }{L},\pm \frac{4\pi }{L},\cdots$ (with i=x,y,z for d=3). Calculation of the density of states in d=1, 2, 3 dimensions is presented in numerous handbooks [30]; here, we use a compact form referring to the particle density on the Fermi level

(14) $$D\left(\varepsilon_{F}\right)=\frac{d}{2}\frac{n(\varepsilon_{F})}{\varepsilon_{F}},$$

where $n\left(\varepsilon_{F}\right)=\int_{0}^{\varepsilon_{F}}D\left(\varepsilon \right)d\varepsilon$ representing the T→0 limit of Equation (9). Substituting $D(\varepsilon_{F})$, given by Equation (14), into Equation (12), we obtain

(15) $$C_{\mathrm{el}}\approx \frac{\pi^{2}d}{6}\;\frac{nk_{B}^{2}T}{\varepsilon_{F}}.$$

Now, taking $\epsilon_{F}=\frac{1}{2}m_{★}v_{F}^{2}$ with the Fermi velocity

(16) $$v_{F}=\frac{1}{\hbar }{\left.\frac{\partial \varepsilon_{k}}{\partial k}\right|}_{k=k_{F}}=\frac{\hbar k_{F}}{m_{★}},$$

and the Fermi wavevector $k_{F}=\sqrt{2m_{★}\epsilon_{F}/\hbar^{2}}$, we further set $v=v_{F}$ in Equation (3), obtaining

(17) $$\kappa_{\mathrm{el}}\approx \frac{\pi^{2}}{3}\;\frac{nk_{B}^{2}T}{m_{★}v_{F}}\ell .$$

It is now sufficient to divide Equations (4) and (17) side-by-side to derive the WF law as given by Equations (1) and (2).

As mentioned above, the result for free Fermi gas is same for arbitrary dimensionality *d*. More careful analysis also shows that the parabolic dispersion of Equation (13) is not crucial, provided that the Fermi surface is well-defined, with an (approximately) constant D(ε)>0 in the vicinity of $|\varepsilon -\varepsilon_{F}|≲k_{B}T$, and that the effective mass $0<m_{★}<+\infty$. In the framework of Landau’s Fermi-liquid (FL) theory, the reasoning can be extended onto effective quasiparticles, and the validity of the WF law is often considered as a hallmark of the FL behavior [31,32].

The suppositions listed above are clearly not satisfied in graphene close to the charge-neutrality point.

### 2.3. The Dirac Gas in Graphene

The relation between thermal conductivity and heat capacity given by Equation (3) holds true for both massive and massless particles. A separate issue concerns the Drude formula (4), directly referring to the effective mass, an adaptation of which for massless Dirac fermions requires some attention.

The Landauer–Büttiker conductivity of ballistic graphene, first calculated analytically employing a basic mode-matching technique [19,20,21] and then confirmed in several experiments [33,34], is given solely by fundamental constants

(18) $$\sigma_{0}=\frac{4e^{2}}{\pi h}.$$

Remarkably, for charge-neutral graphene both the carrier concentration and the effective mass vanish, a finite (and nonzero) value of $\sigma_{0}$ (18) may therefore be in accord with the Drude formula, at least in principle.

To understand the above conjecture, we refer to the approximate dispersion relation for charge carriers in graphene, showing up so-called Dirac cones,

(19) $$E=\pm \hbar v_{F}k.$$

The value of the Fermi velocity $v_{F}\approx 10^{6}\;$ m/s is now energy-independent, being determined by the nearest-neighbor hopping integral on a honeycomb lattice ($t_{0}=2.7\;$eV) and the lattice constant (a=0.246 nm) via

(20) $$\hbar v_{F}=\frac{\sqrt{3}}{2}t_{0}a.$$

Charge carriers in graphene are characterized by an additional (next to spin) quantum number, the so-called valley index. This leads to an additional twofold degeneracy of energy levels, which needs to taken into account when calculating the density of states,

(21) $$D\left(\varepsilon \right)=\frac{2|\varepsilon |}{\pi {\left(\hbar v_{F}\right)}^{2}}.$$

Subsequently, the carrier concentration at T=0 is related to the Fermi energy (and the Fermi wavevector) via

(22) $$n=\int_{0}^{\varepsilon }D\left(\varepsilon^{\prime }\right)d\varepsilon^{\prime }=\frac{\varepsilon^{2}}{\pi {\left(\hbar v_{F}\right)}^{2}}=\frac{k^{2}}{\pi }.$$

In the above, we intentionally omit the *F* index for symbols denoting the Fermi energy and the Fermi wavevector to emphasize that they can be tuned (together with the concentration) by electrostatic gates, while the Fermi velocity $v_{F}$ (20) is a material constant [35].

Despite the unusual dispersion relation, given by Equation (19), the relevant effective mass describing the carrier dynamics in graphene is the familiar cyclotronic mass

(23) $$m_{C}=\frac{\hbar^{2}}{2\pi }\frac{\partial A(\varepsilon )}{\partial \varepsilon }=\frac{\hbar k}{v_{F}},$$

where A(ε) denotes the area in momentum space $\left(k_{x},k_{y}\right)$ bounded by the equienergy surface for a given Fermi energy (ε). It is easy to see that, for a two-dimensional system, with fourfold degeneracy of states, we have $\partial A(\varepsilon )/\partial \varepsilon =\pi^{2}D\left(\varepsilon \right)$; substituting D(ε) given by Equation (21) leads to the rightmost equality in Equation (23). Remarkably, the final result is formally identical with the rightmost equality in Equation (16) for free Fermi gas (albeit now the effective mass, but not the Fermi velocity, depends on the Fermi energy).

Assuming the above carrier density *n* (22), and the effective mass $m_{★}$ (23), and comparing the universal conductivity $\sigma_{0}$ (18) with the Drude formula (4), we immediately arrive to the conclusion that mean-free path for charge carriers in graphene is also energy-dependent, taking the asymptotic form

(24) $$\ell_{\mathrm{eff}}\left(\varepsilon \right)≃\frac{2}{\pi k}=\frac{2\hbar v_{F}}{\pi \varepsilon },\;\mathrm{for}\;\;\;\varepsilon \to 0.$$

Strictly speaking, the ε→0 limit has n→0, i.e., no free charge carriers, and the transport is governed by evanescent waves [6]. The universal value of $\sigma_{0}$ (18) indicates a peculiar version of the tunneling effect appearing in graphene, in which the wavefunction shows a power-law rather then exponential decay with the distance [22], resulting in the enhanced charge (or energy) transport characteristics. Therefore, the mean-free path should be regarded as an effective quantity, allowing one to reproduce the measurable characteristics in the ε→0 limit. Away from the charge-neutrality point, i.e., for $\left|\varepsilon \right|\gg \pi \hbar v_{F}/L$ (with the geometric energy quantization $∼\pi \hbar v_{F}/L$), graphene behaves as a typical ballistic conductor, with $\ell_{\mathrm{eff}}∼L$. We revisit this issue in Section 4, where the analysis starts from actual σ(ε) functions for selected mesoscopic systems, but now the approximation given by Equation (24) is considered as a first.

We further notice that the form of $\ell_{\mathrm{eff}}\left(\varepsilon \right)$ in Equation (24) is formally equivalent to the assumption of linear relaxation time on energy dependence in the Boltzmann equation, proposed by Yoshino and Murata [36].

In the remaining part of this section, we derive explicit forms of the thermal conductivity κ and the Lorentz number L, pointing out the key differences appearing in comparison to the free Fermi gas case (see Section 2.2).

The calculations are particularly simple for charge-neutral graphene (n=ε=0), which is presented first. Although we still can put $v=v_{F}$ in Equation (3), since the Fermi velocity is energy-independent, the constant-density of states approximation applied in Equation (7) in now invalid. (In addition, for T>0, we cannot put $\varepsilon_{F}\gg k_{B}T$ now.) In turn, the expression for heat capacity *C* needs to be re-derived.

For charge-neutral graphene at T>0, contributions from thermally excited electron and holes are identical; it is therefore sufficient to calculate the former:

(25) $$U_{e}\left(T\right)=\int_{0}^{\infty }d\varepsilon D\left(\varepsilon \right)f\left(\varepsilon \right)\;\varepsilon =\frac{2{\left(k_{B}T\right)}^{3}}{\pi {\left(\hbar v_{F}\right)}^{2}}\int_{0}^{\infty }dx\;\frac{x^{2}}{e^{x}+1}.$$

Again, the integral in the rightmost expression in Equation (25) can be expressed via the Riemann zeta function, and is equal to

(26) $$\int_{0}^{\infty }dx\frac{x^{2}}{e^{x}+1}=\frac{3}{2}\zeta \left(3\right)\approx 1.8031.$$

Differentiating Equation (25) with respect to *T*, and multiplying by a factor of 2 due to the contribution from holes in the valence band, we obtain the heat capacity

(27) $$C=\frac{18\;\zeta (3)}{\pi }\;\frac{k_{B}^{3}T^{2}}{{\left(\hbar v_{F}\right)}^{2}}.$$

It remains now to calculate the effective mean-free path *ℓ* to be substituted into Equation (3). We use here the asymptotic form of $\ell_{\mathrm{eff}}\left(\varepsilon \right)$ (24), replacing the $\varepsilon^{-1}$ factor by its overage over the grand canonical ensemble, namely

(28) $${\langle \epsilon^{-1}\rangle }_{T>0}=\frac{\int_{0}^{\infty }d\epsilon D\left(\epsilon \right)f\left(\epsilon \right)\epsilon^{-1}}{\int_{0}^{\infty }d\epsilon D\left(\epsilon \right)f\left(\epsilon \right)}=\frac{12\ln2}{\pi^{2}k_{B}T}.$$

Substituting the above, together with the heat capacity *C* (27), into Equation (3), we get

(29) $$\kappa =\frac{432\;\zeta (3)\ln2}{\pi^{3}h}\;k_{B}^{2}T,$$

and

(30) $$\frac{\kappa }{\sigma_{0}T}=\frac{108\;\zeta (3)\ln2}{\pi^{2}}{\left(\frac{k_{B}}{e}\right)}^{2}\approx 2.7714\times L_{0},$$

with $L_{0}$ being the Fermi-gas result given by Equation (2).

A simple reasoning, presented above, indicates that the κ/σ ratio is significantly enhanced in charge-neutral graphene, comparing to the free Fermi gas. However, the WF law is still satisfied, since the Lorentz number given by Equation (30) is temperature-independent. The situation becomes remarkably different for graphene away from the charge-neutrality point, which is studied next.

Without loss of generality, we suppose μ>0 (the particle hole-symmetry guarantees that measurable quantities are invariant upon μ→−μ). The internal energy U(T) now consists of contributions from majority carries (electrons), with ε>μ, and minority carriers (holes), with ε<μ,

(31) $$\begin{array}{cc} U(T) & =U_{e}+U_{h}\\ \; & =\int_{\mu }^{\infty }d\varepsilon \;D\left(\varepsilon \right)\frac{\varepsilon \;-\;\mu }{\exp\left[\left(\varepsilon \;-\;\mu \right)/k_{B}T\right]+1}+\int_{-\infty }^{\mu }d\varepsilon \;D\left(\varepsilon \right)\frac{\mu \;-\;\varepsilon }{\exp\left[\left(\mu \;-\;\varepsilon \right)/k_{B}T\right]+1}, \end{array}$$

where D(ε) is given by Equation (21). The heat capacity can be written as

(32) $$\begin{array}{cc} C & =\frac{\partial U}{\partial T}=\frac{1}{4k_{B}T^{2}}\int_{-\infty }^{\infty }d\varepsilon \;D\left(\varepsilon \right)\frac{{\left(\varepsilon -\mu \right)}^{2}}{\cosh^{2}\left[\left(\varepsilon -\mu \right)/k_{B}T\right]}\\ \; & =\frac{2k_{B}^{3}T^{2}}{\pi {\left(\hbar v_{F}\right)}^{2}}\;F\left(y\right), \end{array}$$

where we define

(33) $$\begin{array}{cc} F(y) & =\int_{y}^{\infty }\frac{dx\;x^{3}}{\cosh x+1}+y\int_{0}^{y}\frac{dx\;x^{2}}{\cosh x+1}\\ \; & =\frac{\pi^{2}}{3}y-y^{3}+y^{2}\ln\left(2\cosh y+2\right)-8\;{Li}_{2}\left(-e^{-y}\right)-12\;{Li}_{3}\left(-e^{-y}\right), \end{array}$$

with $y=\mu /k_{B}T>0$ and Li${}_{s}\left(z\right)$ being the polylogarithm function [37].

Similarly, the mean-free path can be calculated as

(34) $$\begin{array}{cc} \langle \ell_{\mathrm{eff}}\rangle & =\frac{2\hbar v_{F}}{\pi }{\langle {\left|\epsilon \right|}^{-1}\rangle }_{\mu >0,T>0}=\frac{2\hbar v_{F}}{\pi k_{B}T}\;G\left(y\right), \end{array}$$

where

(35) $$\begin{array}{cc} G(y) & =\ln2\times {\left(\;\int_{y}^{\infty }dx\frac{x}{e^{x}+1}+y\int_{0}^{y}\frac{dx}{e^{x}+1}\;\right)}^{-1}\\ \; & =\ln2\times {\left[\;y^{2}+y\ln\left(e^{-y}+1\right)-{Li}_{2}\left(-e^{-y}\right)-y\ln\left(e^{y}+1\right)+y\ln2\;\right]}^{-1}, \end{array}$$

and $y=\mu /k_{B}T$ again.

**Figure 2:**
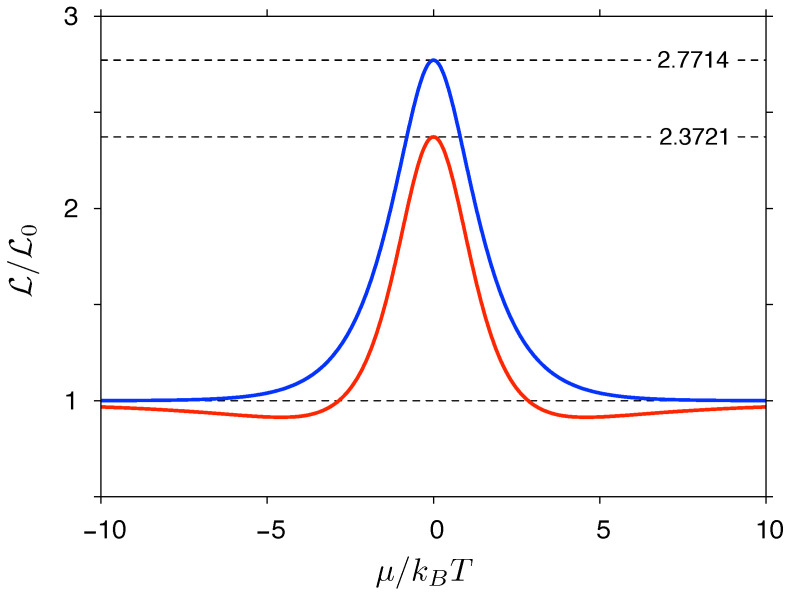
The Lorentz number $L=\kappa_{\mathrm{el}}/\left(\sigma T\right)$ for massless Dirac fermions as a function of the chemical potential. Solid lines represent the approximations given by Equation (36) (blue line) and Equation (39) (red line). Dashed lines (top to bottom) depict the two corresponding μ=0 values, and the value of $L_{0}=\left(\pi^{2}/3\right)\;k_{B}^{2}/e^{2}$ representing the Wiedemann–Franz law restored in the $\left|\mu \right|\gg k_{B}T$ limit.

Hence, the Lorentz number for μ>0 is given by

(36) $$L=\frac{\kappa }{\sigma_{0}T}=F\left(y\right)\;G\left(y\right){\left(\frac{k_{B}}{e}\right)}^{2},$$

with F(y) and G(y) given by Equations (33) and (35). The Lorentz number given by Equation (36) is depicted in Figure 2. It is straightforward to show that in the y→0 limit one obtains the value given by Equation (30) for μ=0; in addition, for y→∞, we have $L\to L_{0}$, restoring a standard form of the WF law for metals. However, for 0<y<+∞, a fixed value of μ (or *n*) corresponds to *y* (and thus L) varying with temperature; namely, the violation of the WL law occurs.

## 3. Landauer–Büttiker Formalism and Simplified Models

### 3.1. The Formalism Essential

In the Landauer–Büttiker description transport properties of a mesoscopic system, attached to the leads, are derived from the transmission-energy dependence T(ε), to be found by solving the scattering problem [38,39,40,41]. In particular, the Lorentz number can be written as [42]

(37) $$L=\frac{\kappa_{\mathrm{el}}}{\sigma T}=\frac{L_{0}L_{2}-L_{1}^{2}}{e^{2}T^{2}L_{0}^{2}},$$

where $L_{n}$ (with n=0,1,2) are given by

(38) $$L_{n}=\frac{g_{s}g_{v}}{h}\int d\varepsilon \;T\left(\varepsilon \right)\left(-\frac{\partial f}{\partial \varepsilon }\right){\left(\varepsilon -\mu \right)}^{n},$$

with $g_{s}=g_{v}=2$ denoting spin and valley degeneracies in graphene, and the Fermi–Dirac distribution function f(ε) given by Equation (8). It is easy to show that energy-independent transmission (T(ε)=const) leads to $L=L_{0}$ (2).

### 3.2. Simplified Models

Before calculating T(ε) directly for selected systems in Section 4, we first discuss basic consequences of some model T(ε) functions for L.

For instance, the linear transmission-energy dependence (i.e., T(ε)∝|ε|) allows one to obtain a relatively short formula for L at arbitrary doping [7], namely

(39) $$\begin{array}{cc} L & =\{\;\frac{\pi^{2}y+y^{3}-12\;{\mathrm{Li}}_{3}\left(-e^{-y}\right)}{\ln\left(2\cosh y+2\right)}\\ \; & \;-{\left[\;\frac{\pi^{2}/3+y^{2}+4\;{\mathrm{Li}}_{2}\left(-e^{-y}\right)}{\ln\left(2\cosh y+2\right)}\;\right]}^{2}\}{\left(\frac{k_{B}}{e}\right)}^{2}, \end{array}$$

with $y=\mu /k_{B}T$. For y=0, the Lorentz number given by Equation (39) takes the value of

(40) $$L\left(0\right)=\frac{9\;\zeta (3)}{2\ln2}{\left(\frac{k_{B}}{e}\right)}^{2}\approx 2.3721\times L_{0},$$

being close to that given in Equation (30). The approximation given in Equation (40) was earlier put forward in the context of high-temperature superconductors also showing the linear transmission-energy dependence [43].

Numerical values of L(y) are presented in Figure 2. Remarkably, L(y) obtained from Equation (36) (blue line) is typically 20–30% higher than obtained Equation (39) (red line). The deviations are stronger near $\left|\mu \right|/k_{B}T\approx 4.5$, where the latter shows broad minima absent for the former. Above this value, L(y) obtained from Equation (36) approaches $L_{0}$ from the top, whereas L(y) obtained from Equation (39) approaches $L_{0}$ from the bottom. In addition, the right-hand side of Equation (36) converges much more quickly to $L_{0}$ for $\left|\mu \right|\gg k_{B}T$ than the right-hand side of Equation (39).

In both cases, the Lorentz number enhancement at the charge-neutrality point (μ=0) is significant, and the violations of the WF law for μ≠0 is apparent. The relatively good agreement between the two formulas is striking: although both derivations have utilized the linear dispersion of the Dirac cones, being link to D(ε) given by Equation (21) in the first case, or to the T(ε)∝|ε| assumption in the second case (see Section 4 for further explanation), only the derivation of Equation (36) incorporates the information about the universal conductivity ($\sigma =\sigma_{0}$). We can therefore argue that the L enhancement occurs in graphene due to the linear dispersion rather then due the transport via evanescent waves (being responsible for $\sigma =\sigma_{0}$ at μ=0).

**Figure 3:**
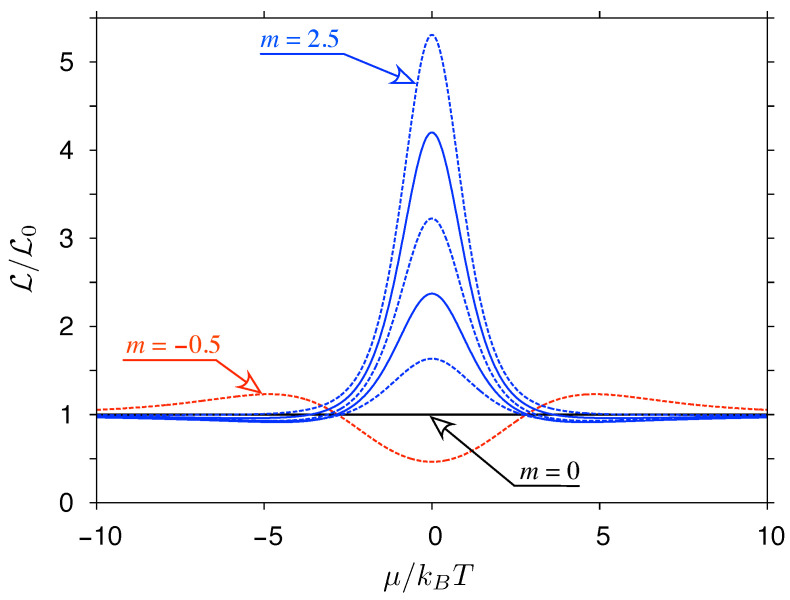
The Lorentz number for model transmission-energy dependence T(ε) given by Equation (41) with *m* varied from −0.5 to 2.5 with the steps of 0.5 displayed as a function of the chemical potential. Solid (dashes) lines mark integer (non-integer) *m*.

We now elaborate possible effects, on the Lorentz number, of toy-models of transmission-energy dependence

(41) $$T\left(\varepsilon \right)\propto {\left|\varepsilon \right|}^{m},\;m>-1,$$

where the proportionality coefficient is irrelevant due to the structure of Equation (37). For some cases, integrals can be calculated analytically, leading, e.g., to $L=L_{0}$ for m=0 (the constant transmission case) or L=L(y) given by Equation (39) for m=1 (the linear transmission-energy dependence). Numerical results for selected values of m=−0.5⋯2.5 are displayed in Figure 3.

The violation of the WF law appears generically for m≠0 away from the charge-neutrality point (i.e., for μ≠0).

**Figure 4:**
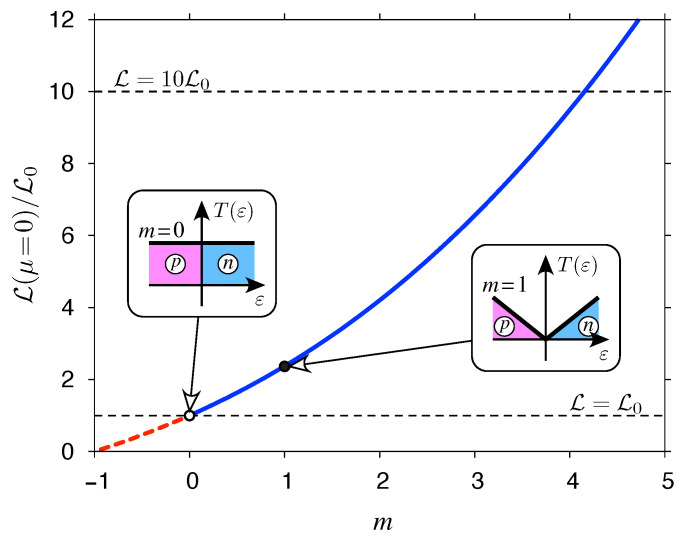
Maximal (solid blue line for m>0) or minimal (dashed red line for −1<m<0) values of the Lorentz number L (reached at μ=0) obtained from Equation (42). [The analytic continuation for m→0 and m→1 is assumed in the righmost expression in Equation (42).] Insets visualize the T(ε) function given by Equation (41) for m=0 and m=1, with contributions from the valence band (p) and the conduction band (n).

For μ=0, the Lorentz number reaches a global maximum (with $L>L_{0}$) if m>0 or a global minimum (with $L<L_{0}$) if −1<m<0. A close-form expression can be derived for both cases, namely

(42) $$\frac{L(\mu \;=\;0)}{{\left(\frac{k_{B}}{e}\right)}^{2}}\;=\frac{\int_{0}^{\infty }dx\frac{x^{m+2}}{\cosh^{2}\left(\frac{x}{2}\right)}}{\int_{0}^{\infty }dx\frac{x^{m}}{\cosh^{2}\left(\frac{x}{2}\right)}}=\frac{2^{m+1}-1}{2^{m+1}-4}\left(m\;+\;1\right)\left(m\;+\;2\right)\frac{\zeta (m\;+\;2)}{\zeta (m)},$$

and is visualized in Figure 4. It is clear that T(ε) models given by Equation (41) may lead to arbitrarily high $L_{\max}$; in particular, the value of $10\;L_{0}$ is exceeded starting from m≈4.1.

Hence, for m>1, the model grasps the basic features of one-dimensional Luttinger liquids, showing both the power-law transmission energy dependence, with nonuniversal (interaction dependent) exponents, and the significantly enhanced Lorentz numbers [12].

On the other hand, the suppression of L is observed for −1<m<0, due to the integrable singularity at ε=0, constituting an analogy with heavy fermion systems [13].

Both the above-mentioned scenarios were described theoretically for quantum dot systems, which may be tuned from the suppression of L due to Breit–Wigner resonance, to the enhancement of L due to Fano resonance [44,45,46].

### 3.3. Gapped Systems

For completeness, we show here how the energy (or transport) gap may enhance the Lorentz number. Instead of T(ε) given by Equation (41), we put

(43) $$T\left(\varepsilon \right)\propto \Theta \left(\left|\varepsilon \right|-\frac{1}{2}\Delta \right){\left(\left|\varepsilon \right|-\frac{1}{2}\Delta \right)}^{m},\;m>-1,$$

where Θ(x) is the Heaviside step function.

For $\Delta \gg k_{B}T$ and Δ≫|μ|, the integrals occurring in Equation (37) can be approximated by elementary functions [47] and for the maximal L (reached for μ=0) we have

(44) $$\frac{L_{\max}}{{\left(\frac{k_{B}}{e}\right)}^{2}}\approx {\left(\frac{\Delta }{2k_{B}T}+m+1\right)}^{2}+m+1.$$

This time, the result given in Equation (44) can be simplified in the m→−1 limit and takes the form of $L_{\max}\approx {\left(\Delta /2eT\right)}^{2}$. Physically, such a limit is equivalent to the narrow band case, namely, $T\left(\varepsilon \right)\propto \delta \left(\varepsilon +\frac{1}{2}\Delta \right)+\delta \left(\varepsilon -\frac{1}{2}\Delta \right)$, with δ(x) being the Dirac delta function.

An apparent feature of Equation (44) is that $L_{\max}$ shows an unbounded growth with a gap (with the leading term being of the order of ∼$\Delta^{2}$), in agreement with the experimental results for semiconductors [11]. Similar behaviors can be expected for tunable-gap systems, such as bilayer graphene or silicene, which are beyond the scope of this work. We only notice that compact formulas, such as Equation (42) for Δ=0 or Equation (44) for $\Delta \gg k_{B}T$, are unavailable if $\Delta ∼k_{B}T$ (which may also by relevant for graphene with a small substrate- or deformation-induced gap). In such a case, numerical integration generically leads to the enhanced $L_{\max}$ (compared to the Δ=0 case) at a fixed *m*.

A different behavior appears near the band boundary, i.e., for μ≈Δ/2 (or μ≈−Δ/2). Assuming $\Delta \gg k_{B}T$ again, we arrive to the limit of an unipolar system, for which only the contribution from majority carries to integrals $L_{n}$ (38) matters. In effect, the Lorentz number can be approximated as

(45) $$L\left(\mu \approx \frac{1}{2}\Delta \gg k_{B}T\right)\approx \left[\frac{J_{2}\left(m;y\right)}{J_{0}\left(m;y\right)}-{\left(\frac{J_{1}\left(m;y\right)}{J_{0}\left(m;y\right)}\right)}^{2}\right]\times {\left(\frac{k_{B}}{e}\right)}^{2},$$

where $y=\left(\mu -\frac{1}{2}\Delta \right)/k_{B}T$ and

(46) $$J_{n}\left(m;y\right)=\int_{-y}^{\infty }dx\;x^{n}\frac{{\left(x+y\right)}^{m}}{\cosh^{2}\left(x/2\right)}.$$

**Figure 5:**
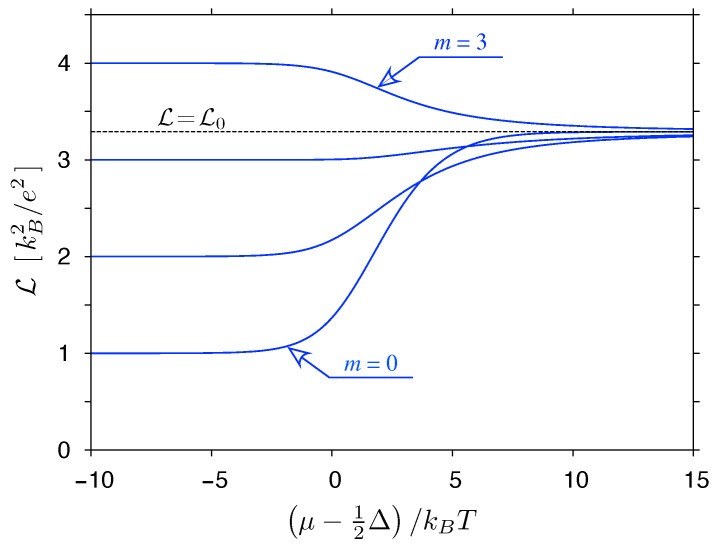
The Lorentz number as a function of chemical potential for the limit of an unipolar system, corresponding to T(ε) given by Equation (43) with $\Delta \gg k_{B}T$ and μ≈Δ/2 (see also Equation (45)). The exponent *m* is varied from 0 to 3 with the steps of 1 (solid lines). Dashed line marks the Wiedemann–Franz value ($L=L_{0}$).

Closed-form expressions for $J_{n}\left(m;y\right)$ are not available; a few numerical examples for m=0⋯3 are presented in Figure 5. Since now $L_{1}\propto J_{1}\neq 0$ (in contrast to the bipolar case studied above), the Lorentz number is significantly reduced and relatively close to $L_{0}$, which is approached for y≫1.

Asymptotic forms of $J_{n}\left(m;y\right)$ can be derived for |y|≫1, namely

(47) Jn(m;y→−∞)≃4ey∫0∞dt(t−y)ntme−t=4ey∑k=0nnk(−y)kΓ(m+k+1),

where Γ(z) denotes the Euler gamma function and

(48) $$\begin{array}{cc} J_{n}\left(m;\;y\;\to \;+\;\infty \right) & ≃y^{m}\int_{-\infty }^{\infty }dx\frac{x^{n}}{\cosh^{2}\left(x/2\right)}\\ \; & =y^{m}\times \left\{\begin{array}{cc} 1, & \mathrm{for}\;\;n=0,\\ 2\left(1\;-\;2^{1-n}\right)\Gamma \left(n\;+\;1\right)\zeta \left(n\right),\; & \mathrm{for}\;\;n⩾1. \end{array}\right. \end{array}$$

Substituting the above into Equation (45), we obtain

(49) $$L\to \left(m+1\right){\left(\frac{k_{B}}{e}\right)}^{2}\;\;\mathrm{for}\;\;\;y\to -\infty ,$$

or

(50) $$L\to \frac{\pi^{2}}{3}{\left(\frac{k_{B}}{e}\right)}^{2}=L_{0},\;\;\mathrm{for}\;\;y\to \infty .$$

Both limits are closely approached by the numerical data in Figure 5 for |y|≳5. In all cases considered, the values of L are now much lower than the corresponding $L_{\max}$ for a gapless model with the same *m* (see Figure 4).

Therefore, it becomes clear from analyzing simplified models of T(ε) that a bipolar nature of the system, next to the monotonically-increasing transmission (the m>0 case), is essential when one looks for a significant enhancement of the Lorentz number L (compared to $L_{0}$).

Both conditions are satisfied for graphene.

## 4. Exactly Solvable Mesoscopic Systems

### 4.1. Transmission-Energy Dependence

The exact transmission-energy dependence T(ε) can be given for two special device geometries in graphene: a rectangular sample attached to heavily-doped graphene leads [19,20,21] and for the Corbino disk [22,23]. Although these systems posses peculiar symmetries, allowing one to solve the scattering problem employing analytical mode-matching method (in particular, the mode mixing does not occur), both solutions were proven to be robust against various symmetry-breaking perturbations [48,49,50,51]. More importantly, several features of the results have been confirmed in the experiments [33,34,52,53] showing that even such idealized systems provide valuable insights into the quantum transport phenomena involving Dirac fermions in graphene.

For a rectangle of width *W* and length *L*, the transmission can be written as [20,22]

(51) $$T\left(\varepsilon \right)=\sum_{n=0}^{\infty }T_{n},$$

where the transmission probability for *n*th normal mode is given by

(52) $$T_{n}={\left[1+{\left(\frac{q_{n}}{k_{n}}\right)}^{2}\sin^{2}\left(k_{n}L\right)\right]}^{-1},$$

with $q_{n}=\pi \left(n+\frac{1}{2}\right)/W$ the quantized transverse wavevector (the constant $\frac{1}{2}$ corresponds to infinite-mass confinement; for other boundary conditions, see [20]),

(53) $$k_{n}=\left\{\begin{array}{cc} \sqrt{k^{2}-q_{n}^{2}}, & \mathrm{for}\;\;k⩾q_{n},\\ i\sqrt{q_{n}^{2}-k^{2}}, & \mathrm{for}\;\;k<q_{n}, \end{array}\right.$$

and $k=|\varepsilon |/(\hbar v_{F})$. The two cases in Equation (53) refer to the contributions from propagating waves ($k⩾q_{n}$, so-called *open channels*) and evanescent waves ($k<q_{n}$).

For the Corbino disk, with its inner ($R_{1}$) and outer ($R_{2}$) radii, we have [22]

(54) $$T\left(\varepsilon \right)=\sum_{j=\pm 1/2,\pm 3/2,\cdots }T_{j},$$

where *j* is the half-odd integer angular momentum quantum number, with a corresponding transmission probability

(55) $$T_{j}=\frac{16}{\pi^{2}k^{2}R_{1}R_{2}}\;\frac{1}{{\left[D_{j}^{\left(+\right)}\right]}^{2}+{\left[D_{j}^{\left(-\right)}\right]}^{2}},$$

where *k* is same as in Equation (53), and

(56) Dj(±)=ImHj−1/2(1)(kR1)Hj∓1/2(2)(kR2)±Hj+1/2(1)(kR1)Hj±1/2(2)(kR2),

with $H_{\nu }^{\left(1,2\right)}\left(\rho \right)$ the Hankel function of the (first, second) kind.

### 4.2. The Conductivity

A measurable quantity that provides a direct insight into the T(ε) function is zero-temperature conductivity

(57) $$\sigma \left(\varepsilon \right)=g_{0}\Omega_{X}T\left(\varepsilon \right),$$

with the conductance quantum $g_{0}=4e^{2}/h$ and a shape-dependent factor

(58) $$\Omega_{X}=\left\{\begin{array}{cc} L/W, & \mathrm{for}\;\mathrm{rectangle},\\ \frac{1}{2\pi }\ln\left(R_{2}/R_{1}\right), & \mathrm{for}\;\mathrm{disk}. \end{array}\right.$$

For T>0, Equation (57) needs to be replaced by $\sigma \left(\mu \right)=e^{2}\;\Omega_{X}L_{0}$, where $L_{0}$ is given by Equation (38) with n=0.

**Figure 6:**
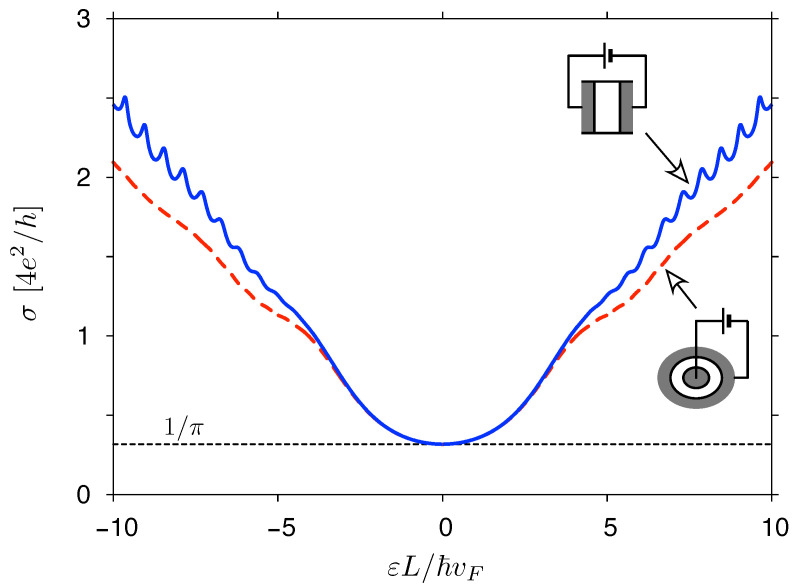
Zero-temperature conductivity as a function of the Fermi energy for a rectangular sample with width-to-length ratio W/L=5 (solid blue line) and the Corbino disk with radii ratio $R_{2}/R_{1}=2$ (dashed red line). Both systems are shown schematically. Dashed black line marks the universal conductivity $\sigma_{0}=\left(4/\pi \right)\;e^{2}/h$.

Numerical results, for T=0, are presented in Figure 6. The data for both systems, displayed versus a dimensionless quantity $\varepsilon L/\hbar v_{F}$ (with $L\equiv R_{2}-R_{1}$ for a disk), closely follow each other up to $|\varepsilon |L/\hbar v_{F}\approx 3$. For larger values of |ε|, the results become shape-dependent and can be approximated, for $\left|\varepsilon \right|\gg \hbar v_{F}/L$, as

(59) $$\sigma \left(\varepsilon \right)\approx g_{0}\Omega_{X}N_{\mathrm{open}}\left(\varepsilon \right){\langle T\rangle }_{\mathrm{open}},$$

where the number of open channels

(60) $$N_{\mathrm{open}}\left(\varepsilon \right)=\left\{\begin{array}{cc} \lfloor kW/\pi \rfloor , & \mathrm{for}\;\mathrm{rectangle},\\ 2\lfloor kR_{1}\rfloor , & \mathrm{for}\;\mathrm{disk}, \end{array}\right.$$

with ⌊x⌋ being the floor function of *x*, and the average transmission per open channel ${\langle T\rangle }_{\mathrm{open}}\approx \pi /4<1$ (for the derivation, see Appendix A). Remarkably, numerical values of σ(ε) for a rectangle with W/L=5 (solid blue line in Figure 6) match the approximation given by Equation (59) with a few-percent accuracy for $\left|\varepsilon \right|≳\;5\hbar v_{F}/L$, whereas for a disk with $R_{2}/R_{1}=2$ (dashed red line) a systematic offset of $\approx \left(1/\pi \right)g_{0}$ occurs, signaling an emphasized role of evanescent waves in the Corbino geometry. This observation coincides with a total lack of Fabry–Perrot oscillations in the Corbino case.

### 4.3. The Lorentz Number

**Figure 7:**
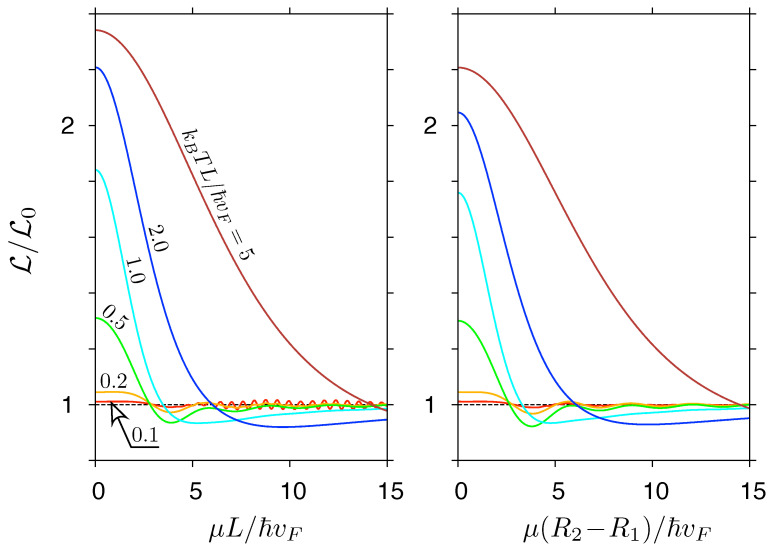
The Lorentz number for a rectangular sample (left) and the Corbino disk (right) displayed as a function of the chemical potential. The temperature, specified in the units of $\hbar v_{F}/\left(k_{B}L\right)\approx 6.67\;$K·μm$\;\times \;L^{-1}$, is varied between the lines and same in both panels. The remaining parameters are the same as in Figure 6.

**Figure 8:**
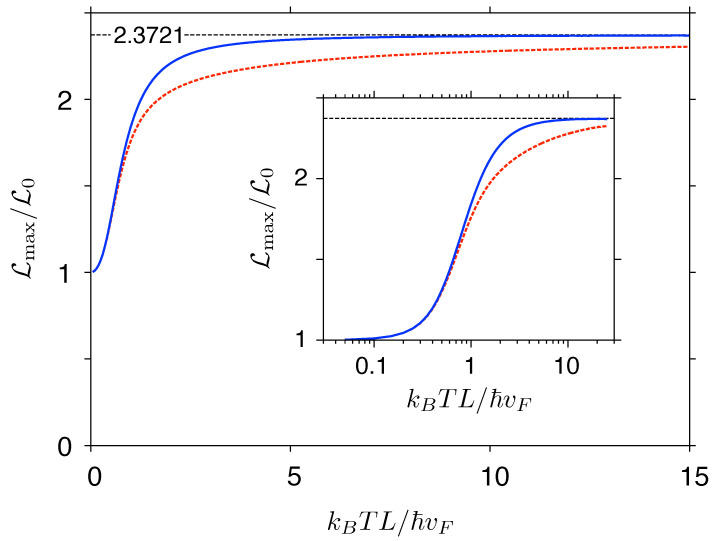
Maximal Lorentz number (corresponding to μ=0) for same systems as in Figure 6 versus temperature. Inset shows the data replotted from the main panel with the abscissa scaled logarithmically. Dashed horizontal line marks the prediction given in Equation (40).

The exact transmission-energy functions T(ε), discussed above, are now substituted into Equation (37) for the Lorentz number. Calculating the relevant integrals numerically, we obtain the results presented in Figure 7 and Figure 8.

Close to the charge-neutrality point, i.e., for |μ|≲max$\left(\hbar v_{F}L^{-1},k_{B}T\right)$, both systems show a gradual crossover (with increasing *T*) from the Wiedemann–Franz regime, with a flat $L\approx L_{0}$, to the linear-transmission regime characterized by L(μ) close to the predicted by Equation (39) (see Figure 7). For higher μ, some aperiodic oscillations of L(μ) are visible if $k_{B}T≲\hbar v_{F}/L$, being particularly well pronounced for a rectangular sample. For higher temperatures, the oscillations are smeared out, leaving only one shallow minimum near $\left|\mu \right|/k_{B}T\approx 4-5$, in agreement with Equation (39).

Maximal values of L for the two systems (reached at μ=0) are displayed, as functions of temperature, in Figure 8. It is clear that a crossover between low and high temperature regimes takes place near $k_{B}T∼\hbar v_{F}/L$ (corresponding to ≈6.67 K for L=1μm): For lower temperatures (and near μ=0), thermally-excited carriers appear in the area where T(ε)≈const (leading to $L\approx L_{0}$), whereas, for significantly higher temperatures, the detailed behavior of T(ε) near ε=0 becomes irrelevant, and the linear-transmission approximation (T(ε)∝|ε|) applies. Remarkably, the convergence to the value given in Equation (40) is much slower (yet clearly visible) in the Corbino disk case, due to a higher (compared to a rectangular sample) contribution from evanescent waves to the transmission away from the charge-neutrality point.

## 5. Conclusions

We calculate the Lorentz number ($L=\kappa_{\mathrm{el}}/\sigma T$) for noninteracting massless Dirac fermions following two different analytic approaches: first, adapting the handbook derivation of the Wiedemann–Franz (WF) law, starting from the relation between thermal conductivity and heat capacity obtained within the kinetic theory of gases, and, second, involving the Landauer–Büttiker formalism and postulating simple model of transmission-energy dependence, T(ε)∝|ε|. In both approaches, the information about conical dispersion relation is utilized, but the universal value of electrical conductivity, $\sigma ∼e^{2}/h$ at ε=0, is referred only in the first approach. Nevertheless, the results are numerically close, indicating the violation of the WF law with maximal Lorentz numbers $L_{\max}/L_{0}\approx 2.77$ and 2.37 (respectively) and $L\to L_{0}=\left(\pi^{2}/3\right)\;k_{B}^{2}/e^{2}$ for high doppings ($\left|\varepsilon \right|\gg k_{B}T$). This observation suggests that violation of the WF law, with $L_{\max}/L_{0}\approx 2-3$, should appear generically in weakly-doped systems with approximately conical dispersion relation, including multilayers and hybrid structures, even when low-energy details of the band structure alter the conductivity. In principle, one can expect similar results for three-dimensional Weyl semimetals [54,55], but experimental separation of the electronic part of thermal conductivity for such systems may be more difficult compared to two-dimensional systems.

Moreover, a generalized model of power law transmission-energy dependence, $T\left(\varepsilon \right)\propto {|\varepsilon |}^{m}$ (with m>−1), is investigated to address the question whether the enhancement of L is due to the bipolar band structure or due to the conical dispersion. Since $L>L_{0}$ shows up for any m>0, and the maximal value grows monotonically with *m*, we conclude that the dispersion relation has a quantitative impact on the effect. On the other hand, analogous discussion of gapped systems, with the chemical potential close to the center of the gap (the bipolar case) or to the bottom of the conduction band (the unipolar case), proves that the bipolar band structure is also important (no enhancement of L is observed in the unipolar case up to m≈2).

Finally, the Lorentz numbers, for different dopings and temperatures, are elaborated numerically from exact solutions available for the rectangular sample and the Corbino (edge-free) disk in graphene, both connected to heavily-doped graphene leads. The results show that L, as a function of the chemical potential μ, gradually evolves (with growing *T*) as expected for a model transmission energy dependence, $T\left(\varepsilon \right)\propto {|\varepsilon |}^{m}$, with the exponent varying from m=0 to m=1. The upper bound is approached faster for the rectangular sample case, but in both cases $L/L_{0}>2$ is predicted to appear for $T≳13\;K\cdot \mu m\times L^{-1}$ with *L* the sample length.

Our results complement earlier theoretical study on the topic [36] by including the finite size-effects and the interplay between propagating and evanescent waves, leading to the results dependent, albeit weakly, on the sample geometry.

## Figures and Tables

**Figure A1:**
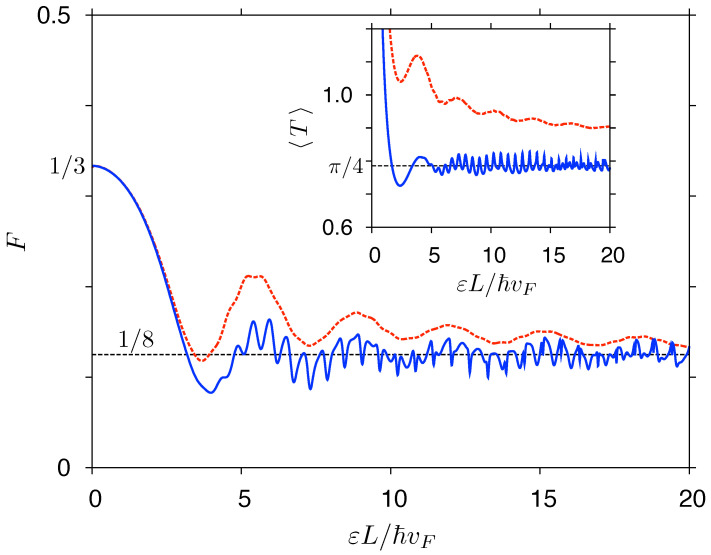
Fermi energy dependence of the Fano factor (main panel) and the average transmission per channel (inset), defined in Equation (A4), for same systems as in Figure 6.

## Data Availability

Numerical datafiles used in the plots are available from the author upon reasonable request.

## Appendix A. Average Transmission per Open Channel and the Enhanced Shot Noise Away from the Dirac Point

In this Appendix, we explain why the average transmission per open channel, occurring in Equation (59) in the main text, is ${\langle T\rangle }_{\mathrm{open}}\approx \pi /4$ instead of 1 (being the value expected for typical ballistic systems). Implications for the shot-noise power are also briefly discussed.

A closer look at Equation (52) for the transmission probability allows us to find out that, for high energy, $k_{n}L\gg 1$ (typically), whereas $q_{n}$ and $k_{n}$, for open channels, are bounded by *k*. Therefore, the average transmission can be approximated by replacing the argument of sine by a random phase 0⩽φ<π, and taking averages over φ and *n* independently,

(A1) $$\begin{array}{cc} {\langle T\rangle }_{\mathrm{open}} & \approx \frac{1}{\pi }\int_{0}^{\pi }d\varphi \int_{0}^{1}dx\frac{1}{1+\frac{x^{2}}{1-x^{2}}\sin^{2}\varphi }\\ \; & =\int_{0}^{1}dx\sqrt{1-x^{2}}=\frac{\pi }{4}, \end{array}$$

where we further introduce a continuous parameterization $x=q_{n}/k$, $\sqrt{1-x^{2}}=k_{n}/k$. In an analogous way, we obtain

(A2) $$\begin{array}{cc} {\langle T^{2}\rangle }_{\mathrm{open}} & \approx \frac{1}{\pi }\int_{0}^{\pi }d\varphi \int_{0}^{1}dx\frac{1}{{\left(1+\frac{x^{2}}{1-x^{2}}\sin^{2}\varphi \right)}^{2}}\\ \; & =\frac{7\pi }{32}. \end{array}$$

The Fano factor [20], quantifying the shot-noise power, can now be approximated, for kL≫1, as

(A3) $$F=\frac{\sum_{n}T_{n}\left(1-T_{n}\right)}{\sum_{n}T_{n}}\approx 1-\frac{{\langle T^{2}\rangle }_{\mathrm{open}}}{{\langle T\rangle }_{\mathrm{open}}}=\frac{1}{8}.$$

The last value in Equation (A3) indicates that shot-noise power in highly-doped graphene is noticeably enhanced compared to standard ballistic systems, which are characterized by F≈0 (as $T_{n}=0$ or 1 for all modes).

Exact results, obtained from first equality in Equation (A3), taking both propagating and evanescent modes into account, are presented in Figure A1. The average transmission, displayed in the inset, is defined as

(A4) $$\langle T\rangle =\frac{T(\varepsilon )}{{\tilde{N}}_{\mathrm{open}}\left(\varepsilon \right)},$$

where ${\tilde{N}}_{\mathrm{open}}\left(\varepsilon \right)$ is calculated from Equation (60) in the main text, in which we omit the floor function (in general, ${\tilde{N}}_{\mathrm{open}}\left(\varepsilon \right)⩾N_{\mathrm{open}}\left(\varepsilon \right)$).

Figure A1 clearly shows that a stronger role of evanescent modes for the Corbino case results in elevated *F* and 〈T〉 (comparing to a rectangle), but a gradual convergence with growing ε to the values given by last equalities in Equations (A3) and (A1) (respectively) is also visible.

It is worth noticing that experimental values of F≈0.15 for highly-doped graphene samples [34] are close, but slightly elevated in comparison to F≈1/8 in Equation (A3). This can be attributed to the tunneling assisted by charged impurities, or other defects, which may amplify the role of evanescent modes also for rectangular samples.
